# Equine Gunshot Euthanasia: Creation of a 3D-Printed Model with Integrated Sensors for Training

**DOI:** 10.3390/ani13162566

**Published:** 2023-08-09

**Authors:** Noël Dybdal, Molly Horgan, Lais Costa, Eric Davis, Steven Lucero, Samantha Nieves, Valerie Quiroz, Kirsten Weberg, John E. Madigan

**Affiliations:** 1 International Animal Welfare Training Initiative, One Health Institute, School of Veterinary Medicine, University of California, Davis, CA 95616, USA; lrcosta@ucdavis.edu (L.C.); ewdavis@ucdavis.edu (E.D.); 2 Department of Medicine and Epidemiology, School of Veterinary Medicine, University of California, Davis, CA 95616, USA; mdhorgan@ucdavis.edu (M.H.); srnievesdvm@gmail.com (S.N.); webergka@gmail.com (K.W.); jemadigan@ucdavis.edu (J.E.M.); 3 Translating Engineering Advances to Medicine (TEAM) Lab, Biomedical Engineering, College of Engineering, University of California, Davis, CA 95616, USA; salucero@ucdavis.edu (S.L.); vgquiroz@ucdavis.edu (V.Q.)

**Keywords:** equine euthanasia, gunshot euthanasia, humane endings, equine welfare

## Abstract

**Simple Summary:**

When conditions make it necessary to end a horse’s life, it must be accomplished in a humane manner. Intravenous administration of an overdose of a concentrated barbiturate anesthetic agent has been the most common method for providing a humane ending in horses. However, due to emerging public safety, barbiturate supply constraints, and environmental reasons, alternative methods for providing humane endings for horses and other large animals are required today. A gunshot correctly delivered to the brain has been shown to be an effective option. It is critical the gun be aimed correctly for the gunshot to destroy specific structures in the brain ensuring instantaneous, permanent unconsciousness and death. Despite the availability of aiming guidelines, studies examining bullet trajectories in animals euthanized by gunshot have identified issues related to incorrect aiming leading to unreliable disruption of the brain structures to ensure a quick and humane death. The availability of a training model and establishment of training guidelines for practitioners to gain proficiency with gunshot euthanasia prior to performing the procedure on animals in the field would correct this welfare issue. This paper describes the development of a 3D-printed, anatomically accurate, economically scalable, and portable model for training in successful humane gunshot euthanasia.

**Abstract:**

Challenges and issues related to the use of pentobarbital euthanasia and disposal of animal remains within the US have recently been reviewed. Environmental and public health challenges increasingly necessitate consideration of alternative methods such as gunshots, an American Veterinary Medical Association (AVMA) “acceptable with conditions” method, for the humane euthanasia of horses. A recent study reported a correctly aimed gunshot provides a humane option for euthanizing horses. However, although aiming guidelines exist, studies examining bullet trajectories in animals euthanized by gunshot have reported that inadequate disruption of the brain is a serious welfare issue. Here, we report the development and production of a portable, reusable, equine gunshot euthanasia training model. Using 3D printing, an anatomically accurate model of an equine head has been developed, with external aiming landmarks and equipped with integrated laser sensors and LED eyes. The laser sensors are embedded in two specific anatomical tracts (pons and medulla) with aiming paths associated with the aiming landmarks to train correct aiming angle. The LED eyes are linked to the laser sensors to provide instant feedback on aiming accuracy. When a beam from a commercially available blue training gun laser travels along the correct aiming path and strikes the sensor inside the head, the lights in the model’s eyes go out and there is an audible signal, providing immediate feedback on the accuracy of the shot. The model facilitates the training of veterinary personnel and first responders in successful gunshot euthanasia, providing instantaneous feedback on the likelihood of a shot causing immediate, humane death in a live animal.

## 1. Introduction

A method of conducting humane euthanasia is critical to ensuring the welfare of horses at their end of life. In recent times, intravenous administration of a barbiturate overdose has been the most common recommended method employed to accomplish a humane ending [1]. However, as it becomes increasingly difficult to dispose of animal carcasses euthanized by a barbiturate overdose, alternative methods that are humane and safe must be employed [2]. A gunshot delivered in a manner that destroys brain regions resulting in instantaneous, permanent unconsciousness is an American Veterinary Medical Association (AVMA) method that is “acceptable with conditions” for euthanizing large animals, including horses, pigs, and cattle [1] (p. 78, 120). The results of a recent study of six different firearm–ammunition combinations, using cadaveric heads placed to simulate a standing horse, have demonstrated that when correctly aimed, each provided a reasonable option for euthanasia of horses [3].

**Figure 1:**
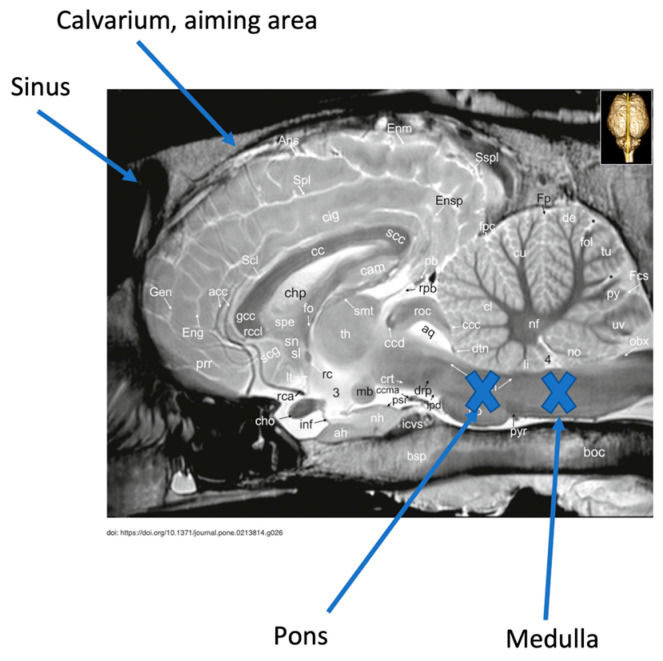
Mid-sagittal MRI image of horse head illustrating the area of brain stem, pons, and medulla oblongata that must be destroyed to achieve humane gunshot euthanasia. Image adapted from Schmidt M, et al. [8].

Guidelines exist describing external landmarks to target to achieve successful gunshot euthanasia [1] (p. 120). Despite these guidelines, studies examining bullet trajectories in animals euthanized by gunshot have demonstrated that inadequate disruption of structures needed to ensure a quick and humane death is an issue [3,4,5]. Breathing and cardiac rhythm are controlled by structures within the medulla oblongata and pons, making it essential to destroy this region of the brainstem to ensure that the horse cannot recover from the state of unconsciousness induced by the initial concussion of the bullet [5,6] (Figure 1). Failed attempts at gunshot euthanasia result in poor animal welfare and cause distress for veterinary personnel, animal owners, and members of the public. A lack of formal training programs for gunshot euthanasia has led to concerns that it is sometimes not performed accurately and is therefore inhumane [7]. To deem a gunshot to be a humane method and ensure horse welfare, it is critical that the bullet trajectory must be accurate. Based on the evidence, there is a need to gain proficiency with gunshot euthanasia prior to performing the procedure on animals in the field.

Simulation-based education, the use of training models to gain proficiency through deliberate practice, is recognized as an effective way to learn without causing harm [9,10]. Development of training models and guidelines for gunshot euthanasia will benefit both horse and human welfare and safety. A durable reusable training model together with training guidelines will provide veterinary professionals, staff, and first responders the opportunity to develop confidence and proficiency in aiming accuracy before attempting gunshot euthanasia in the field. Proficiency developed through practice on the model will minimize the chances of a non-fatal shot and ensure horse welfare. 

Three-dimensional printing, a rapidly evolving technology, is finding expanded availability and use in education. Garcia et al. discussed the technical steps recommended to produce a printed training model. The steps include defining the educational objective, determining the necessary accuracy and resolution of the model, creation of the 3D geometry, optimization of the computer files, and selection of the 3D printer [11]. This paper describes the use of digital imaging (CT scan), Computer-Aided Design (CAD), and 3D printing to produce an anatomically accurate, reusable, battery-operated, portable equine gunshot euthanasia training model. The model’s design provides the user with instantaneous feedback on the accuracy of their shot and likelihood of causing immediate, humane death of a live horse. The model will be used to facilitate the training of veterinary personnel and first responders in successful gunshot euthanasia.

## 2. Materials and Methods

### 2.1. Horse/Computed Tomography Images

No specific authorization from an Institutional Animal Care and Use Committee was required for this study. Computed Tomography (CT) images collected from an adult horse were taken from a repository of normal CT images maintained at the Veterinary Medical Teaching Hospital, University of California-Davis.

### 2.2. Aiming Landmarks (Targets) and Training Firearm

**Figure 2:**
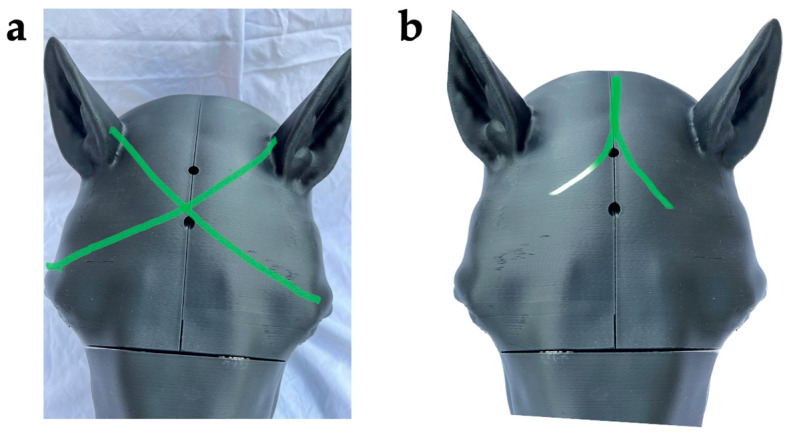
Aiming landmarks. (**a**) At 1–2 inches above the intersection of two lines running from the lateral canthus of the eye to the base of the opposite ear ([1] (p. 120), [12]) or (**b**) along the sagittal crest of the head where the two temporalis muscles form an inverted V [3].

Aiming landmarks on the model include (as described by Shearer and as referenced by Leary et al.) 1–2 inches above the intersection of two lines running from the lateral canthus of the eye to the base of the opposite ear ([1] (p. 120), [12]) and, according to Lund et al., along the sagittal crest of the head where the two temporalis muscles form an inverted V [3] (Figure 2a,b). In the model, achieving the correct aiming angle at the target formed by intersection of the X or the inverted V results in activating the laser sensor embedded in the position of the medulla or the pons, respectively (Figure 1).

Lund et al. reported the successful disruption of the cerebrum, cerebellum, and brainstem of similarly sized equine cadaveric heads using multiple firearm–ammunition combinations, including a semiautomatic 9 mm pistol [3]. Based on this report and the personal experience of the authors (JM, ED) a commercially available laser training pistol, modeled after a 9 mm caliber compact pistol, was selected for use as the model firearm (LaserLyte Trainer Trigger Tyme Laser Compact; https://laserlyte.com/laser-training, accessed on 5 January 2023). 

### 2.3. Development of a Model Head

**Figure 3:**
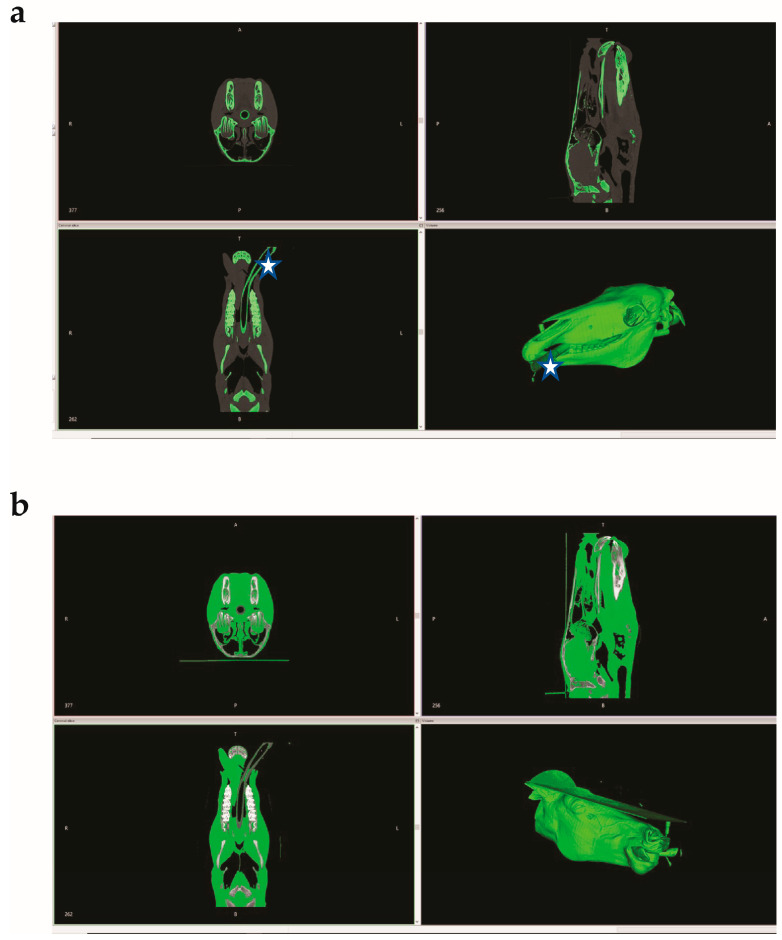
(**a**) Bone (hard tissue) identified and isolated by InVesalius 3 program. Star denotes the misidentified endotracheal tube; (**b**) soft tissue identified and isolated by InVesalius 3 program.

CT images of an equine head were uploaded and processed in the InVesalius 3 program (https://invesalius.github.io/, accessed on 16 May 2023), an open-source software for the reconstruction of CT images. The soft tissues were separated from bone to generate two Standard Tessellation Language files (STL-files). Some features, such as the endotracheal tube, were misidentified by InVesalius 3 (Figure 3a,b). 

**Figure 4:**
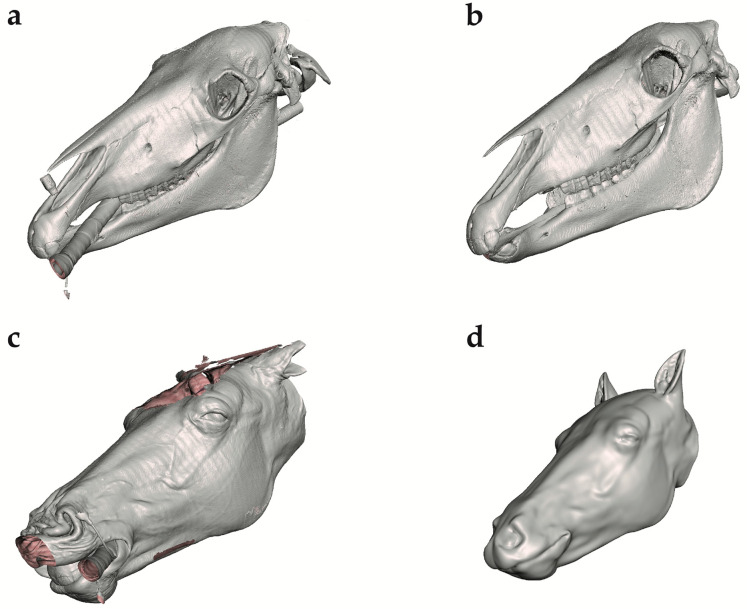
Repair of InVesalius 3 program files in Meshmixer. (**a**) Unrepaired skull file in Meshmixer. (**b**) Repaired skull file in Meshmixer. (**c**) Unrepaired soft tissue file in Meshmixer. (**d**) Repaired soft tissue file in Meshmixer.

**Figure 5:**
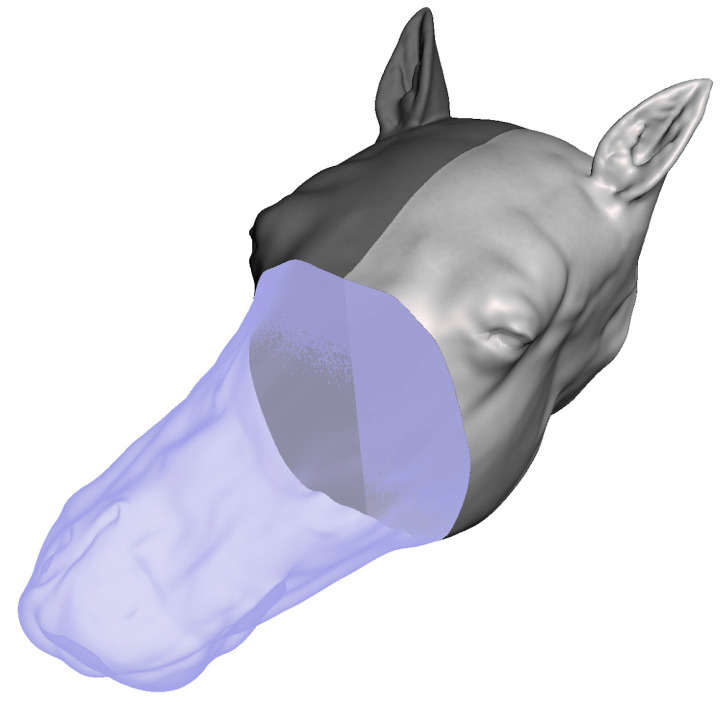
CAD model of head divided into three components to facilitate 3D printing and portability, using Plane Cut command in Meshmixer.

The STL files of the equine head were uploaded into Meshmixer version 3.5.474 (https://Meshmixer.com; accessed on 16 May 2023; free software for working with 3D triangle meshes; Autodesk Inc., San Rafael, CA, USA) to generate a repaired CAD model of the head. The external soft tissue structures were separated from the skeleton and adjustments were made to make the left and right sides of the head symmetrical, as the CT image was taken from an anesthetized horse in recumbency (Figure 4a–d). Any undesired features were removed using commands within the select menu such as Erase and Fill, Discard, Smooth, Transform, and others. To save time and preserve the symmetry, only half of the soft tissue model was repaired and then mirrored across a plane using the mirror command. The soft tissue was divided into three distinct regions using the plane cut command to make assembly, disassembly, repair, and printing easier (Figure 5).

To facilitate 3D printing and portability of the final training model, the CAD model was subdivided into three anatomically distinct regions. Fittings for the 9V battery pack, laser sensors, and LED lights were incorporated into the design with the aid of Inventor version 2022.3 (mechanical design software; Autodesk Inc., San Rafael, CA, USA). The anatomical target area for the laser was set as the medulla oblongata and pons, and the fittings for the LED lights were put into the eyes. NetFabb version Fusion 360 with NetFabb Premium (additive manufacturing, design, and simulation software; Autodesk Inc., San Rafael, CA, USA) was used to automatically repair any remaining mesh defects. When the CAD models were complete, the STL files were prepared for 3D printing using ideaMaker version 4.4.1 alpha (3D slicer program; Raise3D Technologies, Irvine, CA, USA). The three anatomical regions of the head were printed separately using 3D printers (Raise3D N2^®^ and Raise3D N2 Plus^®^; Raise3D Technologies, Irvine, CA, USA).

**Figure 6:**
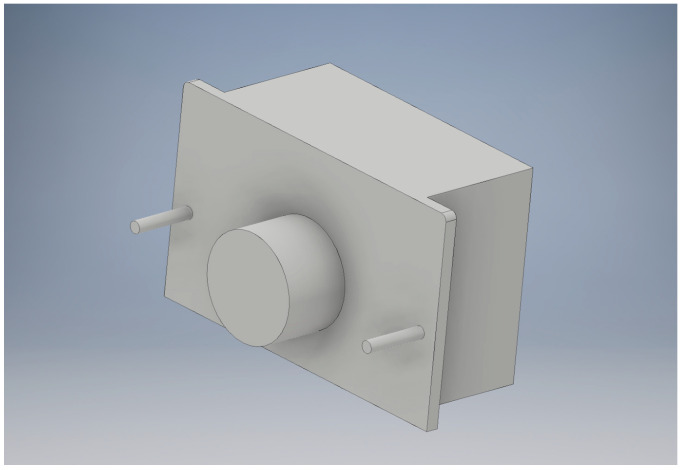
Battery pack negative in Inventor.

It was necessary to create negative models of the electronic components to be embedded in the horse head, including the battery pack, laser sensors, and LEDs (Figure 6). The negative models were subtracted from the soft tissue STL file to generate cavities. Calipers were used to take the measurements of the electronic components that were modeled in the CAD Inventor program. The negative models were designed to be larger than the actual embedded objects to allow a space for clearance and ensure the correct fit. Additional features were also designed onto the negative model, including cylinders on the surface of the model for holes to allow the battery pack to be screwed into the model and an extrusion to allow space for the wires coming out of the battery pack.

**Figure 7:**
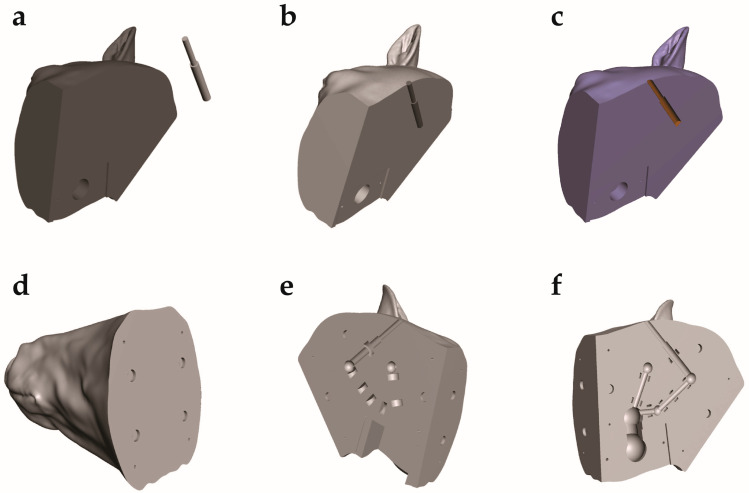
Subtraction of the electronic and assembly components from the STL files. (**a**) Back right of horse head in same scene as sensor in Meshmixer. (**b**) Back right of horse head in same scene as positioned sensor in Meshmixer. (**c**) Back right of horse head with single sensor subtracted. (**d**) Horse muzzle with all components cut out. (**e**) Back left of horse head with all components cut out. (**f**) Back right of horse head with all components cut out (single sensor in example).

The skull STL file was used to properly identify the correct anatomic location for laser sensor placement. Dowel pins and neodymium magnets were used to allow assembly of the three separately printed sections of the model head into a single unit. Cavities for these aligning features were also made in Meshmixer. The cavities for the electronic components including the battery pack, laser sensors, and LEDs were made by creating a negative model in Inventor and then placing it in Meshmixer file. The electronic components were then subtracted from the model head by using a Boolean difference command (Figure 7a–f). 

Any remaining mesh defects were repaired using an automated process in Autodesk NetFabb. 

**Figure 8:**
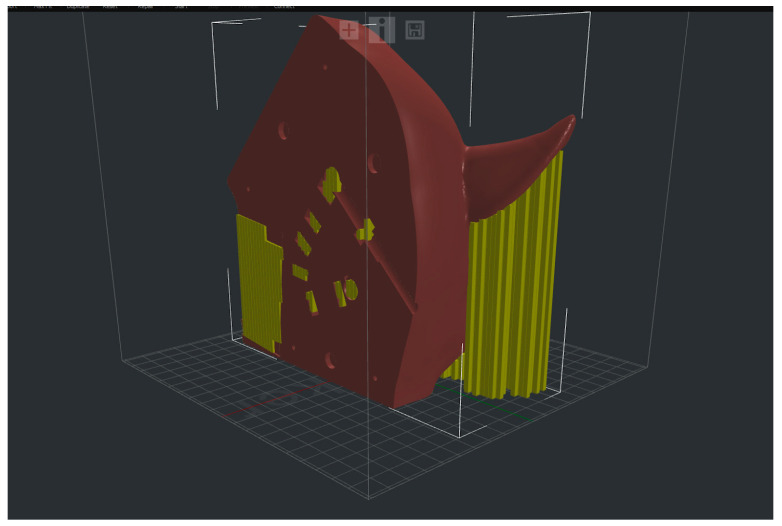
Back left of horse head in ideaMaker with support structure.

The three STL files corresponding to the different anatomical portions of the horse head were imported into ideaMaker. Due to the abnormal shape of the horse head, it was also necessary to incorporate a support structure that would later be removed (Figure 8).

Two 3D printers, a Raise3D (R3D) N2 and a Raise3D N2 Plus, were used to print the head. Both are Fused Deposition Modeling (FDM) 3D printers that use R3D Premium Polylactic Acid (PLA) filament in size 1.75 mm and color black. Each component of the head took approximately 60 h to print and consumed approximately 1 kg of filament.

**Figure 9:**
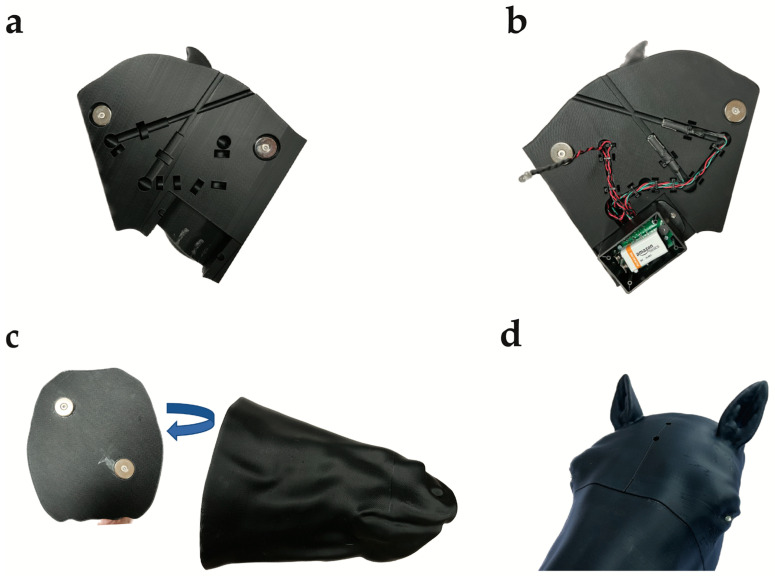
Complete 3D-printed horse head. (**a**) Back left of 3D-printed horse head displaying the aiming channels extending from the external aiming landmarks to the component channel for the laser sensors positioned to simulate the medulla or pons, LED lights, wiring, and battery pack space and neodymium magnets for assembly. (**b**) Back right of 3D-printed horse head displaying aiming channels fitted with laser sensors, wiring from sensors to LED lights and battery pack, and neodymium magnets for assembly in place, with 9 V battery in place. (**c**) Muzzle of 3D-printed horse head with neodymium magnets for assembly. (**d**) Assembled 3D-printed horse head with LED eyes lit.

The three sections of the 3D-printed head were then fitted with neodymium magnets for assembly and the electronics, including the laser sensors, 9-volt battery pack, LED eyes and wiring, to complete the manufacture of the training model (Figure 9a–d). 

## 3. Discussion

**Figure 10:**
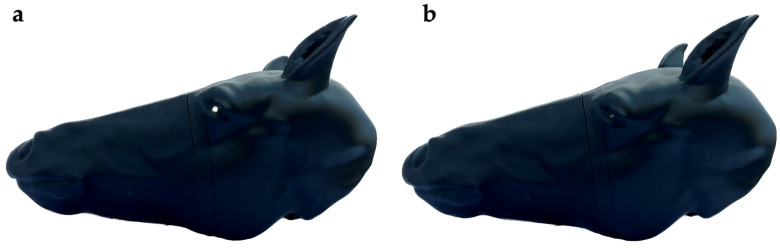
Fully assembled 3D-printed horse head. (**a**) LED eyes lit. (**b**) LED eyes dark after correctly aimed laser training pistol activation of laser sensors embedded in the anatomic locations of the medulla oblongata or pons.

Using digital imaging (CT scan) of a horse head, CAD, and 3D printing, the production of an anatomically accurate, reusable, battery-operated, portable equine gunshot euthanasia training model was described. The model will be used to facilitate the training of veterinary personnel and first responders in successful gunshot euthanasia of horses. In this model, when a laser training firearm is discharged correctly at the external target, the laser sensors embedded in the anatomic location of the medulla oblongata or pons are activated, causing the LED lights in the eyes to go out momentarily and an audible signal to sound, thus providing immediate feedback to the trainee on the likelihood of causing immediate, humane death of a live horse (Figure 10a,b). 

In conducting euthanasia, it is crucial to ensure the welfare of the animal at the end of their life through use of an effective and compassionate method. Environmental and public health concerns related to use of pentobarbital administration have resulted in increased attention on the use of firearms for euthanasia of horses. Previous studies have demonstrated that when accurately aimed, a gunshot to the head provides an instantaneous and humane approach for euthanasia [1,3,4,5,6,13]. A lack of formal training programs for gunshot euthanasia has supported concerns that it is sometimes not performed accurately and is therefore inhumane [14]. Failed attempts at gunshot euthanasia, a source of poor animal welfare, also result in extreme distress for the veterinary personnel, animal owners, and members of the public as well as posing public safety risks.

It is not reasonable to learn, practice, and gain gunshot euthanasia proficiency on living horses, and appropriate horse cadaver practice material is not readily available. In multiple fields, including medicine, simulation technology and training models are acknowledged as a preferred approach to support the mastering of skills through practice (9). In the current communication, we presented the development of a 3D-printed, anatomically accurate, economically scalable, reusable, and portable horse head model for training in successful humane gunshot euthanasia. CT images of an equine head and CAD were successfully used to design and produce this 3D-printed model with integrated laser sensors for use in developing gunshot euthanasia competency. Powered by a 9V battery, the model is equipped with laser sensors integrated into two specific anatomical brainstem locations, the pons and medulla oblongata. The integrated laser sensors communicate with external targeting landmarks and LED lights which serve as the model’s “eyes”. When a beam from a commercially available laser training gun, correctly aimed at the external targets, travels along the correct aiming path and activates the laser sensor inside the head, the lights in the model’s eyes go out and an audible signal is emitted. This training model will provide a valuable tool for any person who expects to perform gunshot euthanasia, allowing the user to practice correct anatomical placement and trajectory of a shot to ensure the brainstem of a horse is hit to cause immediate, humane death.

The described model is based on the CT image of a single horse head. Although the aiming path recommendations remain the same, the development of additional heads based on CT images from a more diverse population including variously sized horses (e.g., ponies and draft horses) or other equids (e.g., donkeys and mules) could be useful. Based on feedback from pilot training sessions, the design of more naturalistic external surface features would also be desirable (e.g., more prominent musculature, addition of a ‘haired’ covering over the plastic). 

The design and production process described here (CT scans, CAD, 3D printing, and anatomical integration of laser sensors) is equally amenable to application for development of training models for additional species, such as cattle, sheep, and pigs, in which gunshot euthanasia is also performed. As with horses, it is critically important to animal welfare that operators performing gunshot euthanasia are proficient in proper targeting and would be supported by opportunities for training [13]. In addition, the 3D-printed training model process could also be used to develop training models to support in the use of captive-bolt shot for single-step euthanasia as recently described by Gilliam et al. [15].

Although this 3D-printed horse head was developed and produced in an academic environment, 3D printing is now widely available commercially. A Google search of “3D printed anatomical models” returned 13,600,000 results and evidence of widely available commercial sources for bespoke model creation (3D-printed anatomical models; searched on 24 July 2023).

While this model will be a useful training tool for developing confidence and learning the best placement of a shot, other factors not encompassed by the model must be included when developing training guidelines for performing successful and safe gunshot euthanasia. These factors include, but are not limited to, personnel well-trained in firearm safety, the type of firearm and ammunition available, and capability of confirming death by checking for a heartbeat, pulse, respirations, and/or absence of reflexes. In addition, the breed and individual variations in anatomy mean that this model may not be exactly representative of all equids. 

## 4. Conclusions

In summary, this study describes the development of a non-cadaveric, anatomically accurate, 3D-printed, and portable horse head model for training in effective and humane equine gunshot euthanasia. This model is equipped with laser sensors and defined tracts to guide training in the correct aiming to cause an immediate and humane death. Expansion of this model to account for different types of firearms and bullets, and for use in other large animal species such as cattle, sheep, and pigs can provide a robust and portable training system that will facilitate the training of veterinary personnel and first responders in successful gunshot euthanasia and ensure the welfare of these animals at the end of their life.

## Data Availability

No additional data generated. Data sharing is not applicable to this communication.
